# Prehospital stroke triage in Denmark: a retrospective observational study of emergency medical services handling confirmed stroke cases not admitted directly to a stroke unit

**DOI:** 10.1186/s13049-026-01579-4

**Published:** 2026-02-13

**Authors:** Astrid Merete Blomberg Drejøe, Magnus Glerup, Troels Wienecke, Jonathan Wenstrup, Felix Nicolai Raben-Levetzau, Heidi Shil Eddelien, Stig Nikolaj Fasmer Blomberg, Helle Collatz Christensen

**Affiliations:** 1 Emergency Medical Services, Prehospital Center, Region Zealand, Ringstedgade 61, 14th floor, Naestved, DK-4700 Denmark; 2 Faculty of Health and Medical Sciences, University of Copenhagen, Copenhagen, Denmark; 3 Department of Neurology, Zealand University Hospital, Roskilde, Denmark; 4 Department of Brain and Spinal Cord Injuries, Rigshospitalet Glostrup, University Hospital of Copenhagen, Copenhagen, Denmark; 5 Department of Neurology, Neurovascular Research Unit, Copenhagen University Hospital-Herlev Gentofte, Copenhagen, Denmark; 6 Department of Health Science and Technology, Aalborg University, Aalborg, Denmark

**Keywords:** Stroke, Triage, Stroke units, Emergency responders, Telemedicine

## Abstract

**Background:**

Early identification and correct triage of stroke in the prehospital setting are essential for timely treatment and improved outcomes. In Region Zealand, Denmark, ambulance personnel are instructed to consult a neurologist when stroke is suspected to facilitate direct admission to a specialised stroke unit. However, little is known about what happens when emergency medical service (EMS) dispatchers correctly suspect stroke, but the patient is not admitted directly to a stroke unit. This includes whether a consultation with a neurologist occurs and on what basis patients are rejected. The primary aim was to determine how often ambulance personnel consulted with a neurologist and examine documented reasons for not referring patients directly to a stroke unit. The secondary aim was to describe symptom presentation in these patients.

**Methods:**

This retrospective observational study linked prehospital ambulance records, Computer-Aided Dispatch data, and the Danish Stroke Register. The prehospital medical record by ambulance personnel was reviewed for each case to examine the decision-making during ambulance responses using a predefined instrument. The study included patients ≥ 18 years who were initially suspected of having a stroke by the EMS dispatcher, were not transported directly to a stroke unit, and a stroke diagnose in the Danish Stroke Register corresponding to the same clinical event from January 2021 to September 2024 in Region Zealand, Denmark. Descriptive statistics were used to summarise findings.

**Results:**

Of the 680 patients included, 583 (86%) were consulted with a neurologist. Patients consulted with a neurologist were older (median 77 vs. 74 years; p = 0.021) and more often exhibited typical stroke symptoms such as paresis and aphasia. Reasons for neurologist rejection were documented in 201 out of 583 consulted patients (34%) and included being outside the thrombolysis time window (19%), contraindications to thrombolysis (15%), few or atypical stroke symptoms (14%), or lack of suspicion of stroke (12%).

**Conclusions:**

While most patients were consulted with a neurologist, a notable proportion were not despite instructions. Reasons for rejection was documented in 34% of patients and included being outside the thrombolysis time window, contraindications, or atypical symptoms. These findings highlight the need to review triage instructions.

## Background

Little is known about what happens when emergency medical services (EMS) dispatchers correctly suspect stroke, but the patient is not directly admitted to a stroke unit, particularly regarding whether a neurologist is consulted and why patients are rejected.

Stroke is a disruption in cerebral blood flow that may result in permanent neurological deficits, disability, or death [1, 2]. It is one of the leading causes of mortality and disabilities in most western countries [3]. In Denmark, approximately 12,000 people suffer a stroke annually. Of these, about 9% die within the first 30 days [4]. Haemorrhagic, ischemic stroke, and transient ischemic attack (TIA) are all considered time-critical conditions [5] and admission to specialized stroke units is associated with improved survival and functional outcomes. These benefits extend beyond acute reperfusion therapy and are attributed to factors such as coordinated multidisciplinary care, early initiation of rehabilitation, structured secondary prevention, and continuous neurological monitoring [5, 6]. In Region Zealand, Denmark, all patients with suspected stroke are therefore intended to be assessed for admission to the regional stroke unit irrespective of eligibility for thrombolytic treatment. According to regional protocol ambulance personnel must consult an on-call neurologist in all suspected stroke cases before transport decisions are made. Direct admission to the stroke unit is therefore contingent on neurologist assessment regardless of symptom duration or eligibility for reperfusion therapy.

Delays in stroke treatment is often due to limited public awareness [3, 7] or due to EMS dispatchers failing to identify a considerable number of stroke cases, [8–10] suggesting that even trained health professionals can face challenges in identifying symptoms of stroke. To our knowledge, little is known about what happens after stroke is correctly recognized by EMS dispatchers, but the patient is not admitted directly to a stroke unit. One possible explanation for the gap is reassessment done by the ambulance personnel or neurologist. If that is the case, there is a need to evaluate the basis for this reassessment. The literature offers limited insight into whether neurologists are consulted with or what criteria underlie these decisions.

The aim of this study was to examine the prehospital characteristics of patients with confirmed stroke who were not transported directly to a stroke unit despite initial recognition as suspected stroke by EMS dispatchers. The primary aim was to describe the frequency of neurologist consultation and to characterize the documented prehospital reasons for non-direct referral to a stroke unit. The secondary aim was to describe the documented prehospital symptom presentation in this patient group.

## Methods

### Study design

This retrospective, observational, register-based study linked data from prehospital ambulance records with data from the Danish Stroke Register [11] and emergency call data from first of January 2021 to first of September 2024. The prehospital medical record written by ambulance personnel [12] was reviewed for each case to explore the decision-making during ambulance responses.

### Setting

The study was conducted in Region Zealand in Denmark, covering approximately 821,000 residents across 7,273 square kilometres [13]. Healthcare services, including EMS services, is tax-funded and provided free of charge [14]. Stroke unit care and thrombolysis treatment is centralized in one primary stroke centre, located at a university hospital. Regional centralization has placed the stroke unit separate from the emergency departments, and patients with suspected stroke should therefore bypass the emergency department and be taken directly to the stroke unit.

### Prehospital response setting for a patient with stroke

In total 58 ambulances were in operation in Region Zealand [15]. Ambulance personnel consist of two professionals, one of whom is at paramedic level or higher [16].

According to the regional prehospital stroke protocol, all patients with suspected acute stroke must be discussed with the on-call neurologist at the stroke unit. This applies regardless of symptom severity, symptom resolution prior to ambulance arrival, or eligibility for thrombolytic treatment. The on-call neurologist determines whether the patient should be admitted directly to the regional stroke unit or referred to a local hospital for assessment. Prehospital Stroke Score (PreSS) is a structured decision support tool to assist the neurological assessment when stroke is suspected. PreSS consists of two parts, where Part 1 supports identification of stroke or TIA and Part 2 is used to identify large-vessel occlusion [17]. Use of PreSS is recommended in the protocol but not mandatory and does not replace clinical judgment or neurologist consultation. PreSS was introduced in July 2021 and was not used consistently to allow any meaningful interpretation.

### Participants

The study included all patients aged ≥ 18 years who were initially suspected of having a stroke by the EMS dispatcher, were not transported directly to a stroke unit, and were registered in the Danish Stroke Register with a confirmed stroke corresponding to the same clinical event.

Dispatcher suspected stroke was used to identify a clinically coherent subgroup in which stroke was the primary working diagnosis at the time of emergency call. Patients with confirmed stroke who were not initially suspected by the dispatcher were therefore not included. Patients transported directly to the stroke unit were excluded, as the objective of this study was to examine the subgroup of patients with confirmed stroke who were not directly admitted. Patients were included from first of January 2021 to first of September 2024.

### Data sources

The study linked data from prehospital ambulance records with data from the Danish Stroke Register [11] and emergency call data with Computer-Aided Dispatch (CAD) system. Patient data were linked across data sources using the Civil Personal Register (CPR) number, a unique personal identifier used in all Danish health records [18]. The CAD system, operated by the EMS dispatchers, is used to log and manage emergency calls including ambulance allocation, triage priority, the diagnosis suspected by the dispatcher, and time-stamped information on call intake. Call times were categorized into day (07:00–15:00), evening (15:00–23:00), and night (23:00–07:00).

The Danish Stroke Register was established in 2003 along with implementation of national stroke care guidelines. All stroke departments are required to report data to the Danish Stroke Register, covering both haemorrhagic and ischemic strokes [11]. The register’s completeness is reported to be > 90% [11].

A nationwide electronic prehospital record has been used by the ambulance personnel since 2015, and all patients are assessed by EMS dispatchers using Danish Index, a triage tool to assess urgency [16]. The prehospital record includes objective findings, vital signs, medication use, and a medical record. Defined data from the medical record is forwarded to the hospital record [16].

### Data entry form and interrater reliability

An initial review of ten prehospital records was performed to identify frequently documented relevant data points to make a data entry form in Research Electronic Data Capture (REDCap) [19]. Two independent reviewers, then used the form to extract data from twenty additional records with focus on the notes written by the ambulance personnel. Interrater reliability was measured using Cohen’s Kappa for each data point in the form. The form was refined in response to the results, and the reviewers repeated the process twice on new records to achieve a minimum Kappa value of 0.8 across all data points, indicating a strong level of agreement between reviewers [20]. The documented reason for not referring a patient to the stroke unit was extracted directly from the prehospital record, and the original wording was kept unchanged to avoid interpretation bias. Reasons were grouped into broader categories or reported separately when a specific reason appeared frequently.

### Statistics

Descriptive statistics were used to summarize the data. Categorical variables were presented as absolute numbers and percentages, with percentages calculated based on available data.

Age was reported as median and first to third quartile (Q1, Q3). Cases were categorized according to whether a neurologist had been contacted in the secondary analysis. The Wilcoxon rank-sum test was used to compare age between groups. Comparisons of categorical variables, including sex, adherence to the time window, and time of emergency call, were performed using either the chi-squared test or Fisher’s exact test, as appropriate. Missing data are reported as counts and percentages in the tables, and no imputation was conducted to preserve interpretability and transparency of the data. All analyses were performed using RStudio (version 4.4.2).

### Ethics

According to the Danish Act on Research Ethics Review of Health Research Projects (Act no. 593 of 14 July 2011, §14(2)), ethical approval is not required for register-based studies that do not involve human biological material. The study was registered and approved by the Danish Data Protection Agency (EMN-2025–07435).

## Results

**Fig. 1:**
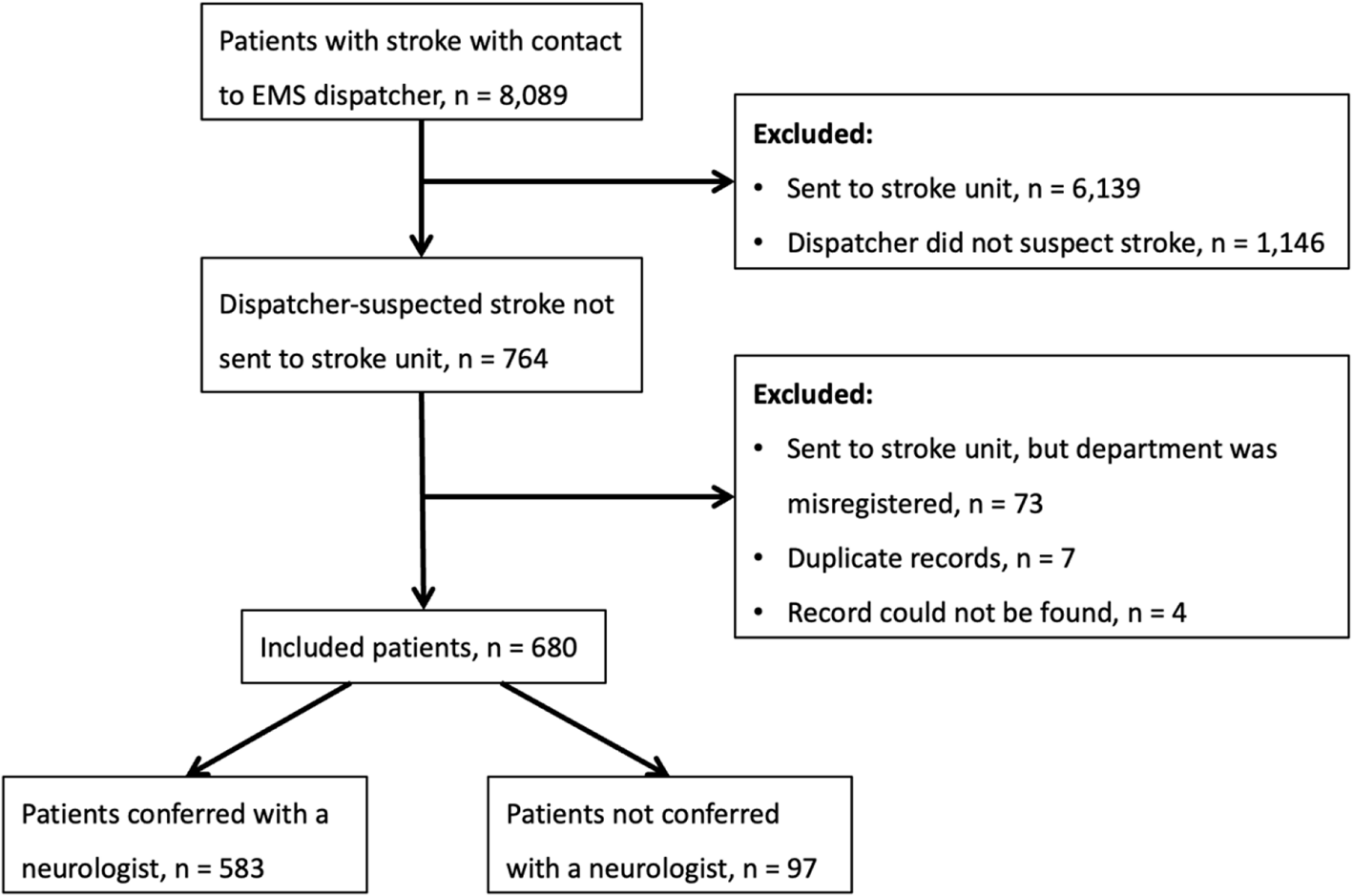
Flowchart of patient inclusion and exclusion from first of January 2021 to first of September 2024. *EMS* Emergency Medical Services

A total of 8,089 patients had a confirmed diagnosis of stroke and contact with an EMS dispatcher during the study period. Of these, 6,139 were initially suspected of having stroke by the EMS dispatcher and were directly transported to the stroke unit and therefore excluded. An additional 73 patients were excluded, since they had been transported to the stroke unit directly, but the department had been incorrectly registered in the prehospital record. Seven duplicate records and four cases where the prehospital record could not be retrieved were also excluded. After exclusion, a total of 680 patients who called the 1–1–2 number at the EMS with a subsequent confirmed stroke were included (Fig. 1). The median age was 76 years (Q1, Q3 = 68, 83) and 46% were women. Emergency calls were most frequently made in the evening, followed by day, and night. When the ambulance arrived, patients had a relative present documented in 34% of cases, and a healthcare professional present documented in 13% of cases, e.g. in a nursing home. Symptom onset was documented by the ambulance personnel in 75% of cases. The most frequently recorded symptoms were paresis, aphasia, sensory disturbances, and dizziness (Table 1).

**Table 1:** Baseline characteristics taken from the prehospital medical record, Region Zealand, Denmark (January 1 st, 2021, to September 1 st, 2024)

*Characteristics*	*Patients, n* = *680*			
***Age, years (median, Q1, Q3)***	76 (68, 83)			
***Female sex, N (%)***	310 (46)			
***Time of day for 1–1–2 call, N (%)***	Day	Evening		Night
	264 (39)	391 (58)		25 (4)
***Weekday of the call, N (%)***				
Monday	88 (13)			
Tuesday	102 (15)			
Wednesday	129 (19)			
Thursday	90 (13)			
Friday	95 (14)			
Saturday	79 (12)			
Sunday	97 (14)			
Comorbidity documented*, N (%)				
* Hypertension*	277 (41)			
* Heart disease*	227 (33)			
* Prior stroke*	196 (29)			
* Diabetes*	115 (17)			
* Cancer*	50 (7)			
* Psychiatric disease*	42 (6)			
* KOL*	40 (6)			
* Dementia*	29 (4)			
* Epilepsy*	14 (2)			
* No disease mentioned*	120 (18)			
***Scene details documented, N (%)***	**Yes**		**Missing**	
*Relatives present at ambulance arrival*	194 (34)		114 (17)	
*Healthcare professional present at ambulance arrival*	88 (13)		9 (1)	
*Debut of symptoms considered*	507 (75)		4 (1)	
***Symptoms documented, N (%)***	**Yes**		**Missing**	
*Paresis*	272 (58)		210 (31)	
Paresis location				
* Face*	155 (57)			
* Extremities*	159 (59)			
Aphasia	272 (68)		279 (41)	
Sensory disturbance	121 (47)		421 (62)	
Dizziness	169 (79)		467 (69)	
Fall recorded	52 (75)		611 (90)	
Impaired consciousness	31 (5)		40 (6)	

^*^One patient can have multiple comorbidities, so percentages exceed 100%

### Primary analysis

In total, 583 patients (86%) were consulted with a neurologist at a stroke unit (See Table 2), while 97 patients (14%) were not. A significant age difference was observed between the two groups. Patients consulted with a neurologist had a median age of 77 years (Q1, Q3 = 68–83), compared with 74 years (Q1, Q3 = 65–79) among those who were not (p = 0.021). No significant differences were found regarding sex, time of emergency call, or whether the patient was within the thrombolysis time window.

**Table 2:** Characteristics of patients stratified by whether a neurologist was consulted with and reason for rejection as stated in the prehospital record, Region Zealand, Denmark (January 1 st, 2021, to September 1 st, 2024)

*Characteristics*	*Patients consulted with a neurologist* *(n* = *583, 86%)*	*Patients not consulted with a neurologist (n* = *97, 14%)*	*P-value*
***Age, years (median, Q1, Q3)***	77 (68, 83)	74 (65, 79)	.021**
***Sex, n (%)***			1.000
* Female*	266 (86)	44 (14)	
* Male*	317 (86)	53 (14)	
In time window for thrombolysis*, yes (%)	331 (86)	54 (14)	.635
***Time of day for call, n (%)***			
* Morning*	24 (96)	1 (4)	.237
* Noon*	227 (86)	37 (14)	.971
* Evening*	332 (85)	59 (15)	.546
***Time of week for call, n (%)***			
* Weekday*	455 (85)	80 (15)	.324
* Weekend*	128 (88)	17 (12)	.324
Reason for rejection as stated in prehospital record, n (%)***			
* No reason stated*	382 (66)	N/A	
* Time window pased or unknown debut of symptoms*	39 (19)	N/A	
* Contraindication for thrombolysis*	31 (15)	N/A	
* Few or atypical symptoms*	29 (14)	N/A	
* No suspicion of stroke*	24 (12)	N/A	
* Scheduled for a consultation at the Stroke unit, but later*	16 (8)	N/A	
* Suspicion of infection*	15 (8)	N/A	
* Suspicion of a bleed*	10 (5)	N/A	
* Suspicion of transient ischemic attack*	8 (4)	N/A	
* Dizziness, dehydration, confusion, blood sugar level, or migraine*	8 (4)	N/A	
* Patient too sick for thrombolysis*	7 (4)	N/A	
* Seizure activity*	5 (3)	N/A	
* Other reason stated*	9 (5)	N/A	

^*^Data from the Danish Stroke Register

^**^Significant p-value

^***^More than one reason could be recorded per patient, so percentages exceed 100%

Reasons for neurologist rejection were documented in 201 cases (34%), while 382 records (66%) lacked documentation. The most frequently stated reasons for rejection to the stroke unit included that the time window had passed (n = 39; 19%), presence of contraindications for thrombolysis (n = 31; 15%), few or atypical symptoms (n = 29; 14%), and a lack of stroke suspicion by the neurologist (n = 24; 12%). Less common reasons included suspected TIA (n = 8; 4%), inebriation (n = 3), suspected eye conditions (n = 2), and capacity limitations at the stroke unit (n = 1). Four patients declined hospital admission and were therefore not consulted with a neurologist.

Sixteen patients (8%) were scheduled for a later appointment at the stroke unit after consultation with the on-call neurologist. The patients were initially left at home by the ambulance and subsequently had to arrange their own transport to the stroke unit. The reason for this was not documented in the prehospital records.

### Secondary analysis

**Fig. 2:**
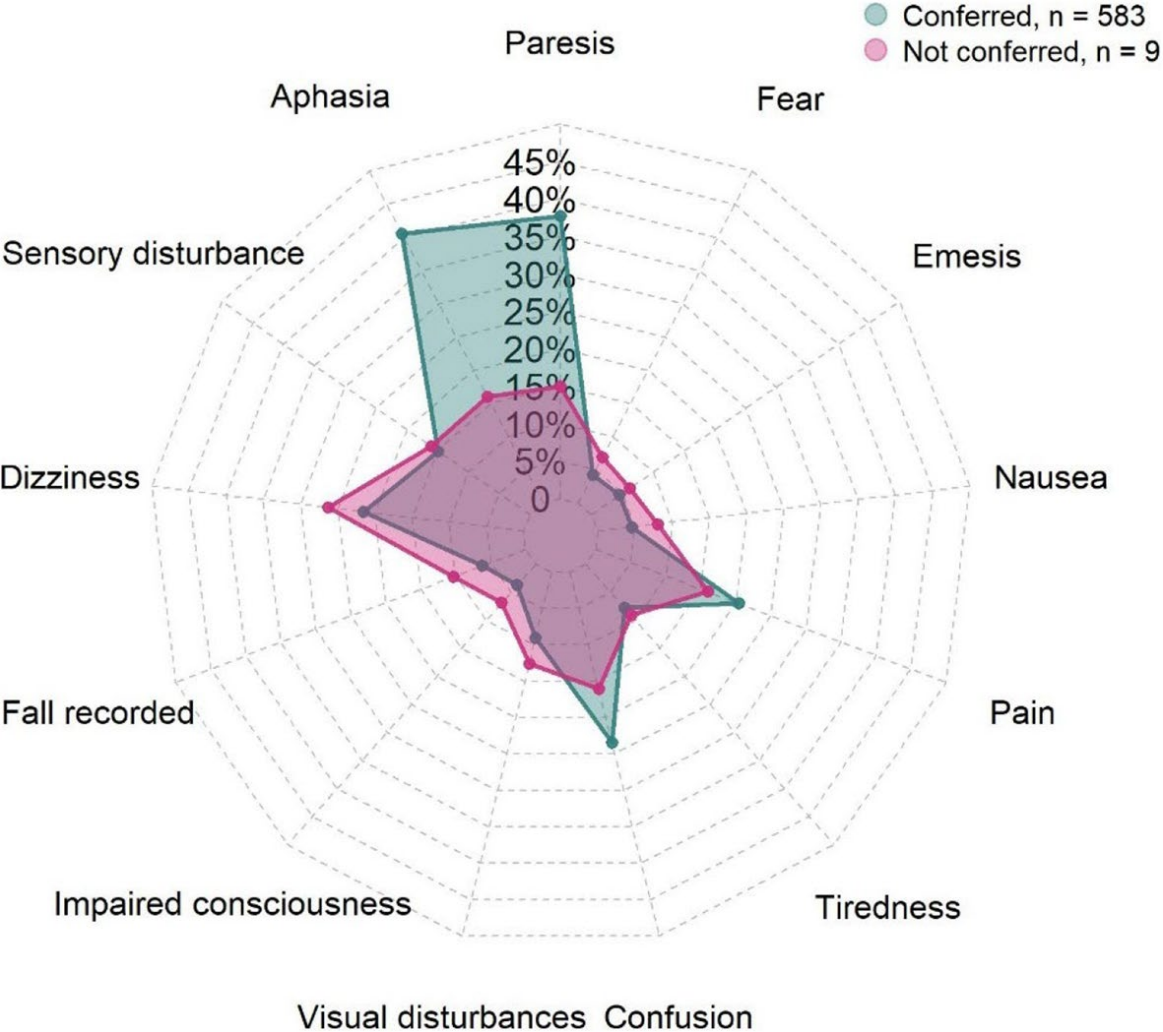
Distribution of symptoms among patients who were consulted with a neurologist compared to those who were not consulted with a neurologist, Region Zealand, Denmark (first of January 2021 to first of September 2024). The spider plot displays the percentages of patients (X-axis) reporting each symptom in the two groups

Among the 680 patients, 583 were consulted with a neurologist and 97 were not. Documented symptoms differed between the two groups (Fig. 2). Aphasia and paresis were substantially more frequent in the consulted group. Confusion also appeared somewhat more frequent in the consulted group. In contrast, most other symptoms, such as sensory disturbances, dizziness, falls, visual problems, impaired consciousness, tiredness, pain, nausea, emesis, and fear, occurred at comparable rates in both groups, with only slight shifts that did not suggest a strong directional trend. (Fig. 2).

## Discussion

### Summary

This retrospective study examined patients with a confirmed stroke who were initially suspected of having a stroke by an EMS dispatcher but were not transported directly to a stroke unit. Consultation with an on-call neurologist was documented in 86% of prehospital records. Neurologists’ reasons for rejecting direct admission were only documented in 34% of prehospital medical records and most frequently included being outside the therapeutic time window, unclear symptom onset, and contraindications to thrombolysis. In other cases, stroke was not suspected by the neurologist due to vague and atypical symptoms or alternative suspected diagnoses. Interestingly, patients who were consulted with a neurologist exhibited a higher frequency of typical stroke symptoms such as paresis and aphasia recorded in the medical record, raising the question of why these classic presentations did not always result in direct admission to the stroke unit. One possible explanation is that these symptoms may have been interpreted as pre-existing sequelae in patients already familiar with such deficits, leading to triage toward the local emergency department rather than the stroke unit. This underscores the inherent complexity of prehospital stroke assessment.

According to the regional prehospital stroke protocol, all patients with suspected stroke should be discussed with a neurologist, who is responsible for the final decision. This structure is designed to place the decision in specialized hands and to balance timely access to stroke unit care against the risk of overwhelming the unit with false positive cases. Given the large number of patients initially suspected of stroke by dispatchers, neurologist consultation plays an important role in sorting out false positive cases prior to stroke unit admission. As a consequence, a proportion of patients with initial stroke suspicion will not be referred directly to the stroke unit following reassessment. The present findings should be interpreted in light of this decision structure, which prioritizes specialized evaluation while accepting the inherent trade-off between sensitivity and specificity in prehospital stroke triage.

Among patients who were not admitted directly to the stroke unit, the most frequently documented reasons were that the time window for thrombolysis had passed or that symptom debut was unknown, and the presence of contraindications to thrombolytic treatment. These findings suggest that eligibility for thrombolysis may have played a disproportionate role in prehospital triage decisions. Admission to a dedicated stroke unit is supported by strong evidence independent of thrombolytic treatment, with benefits attributed to specialized multidisciplinary care, continuous neurological monitoring, early secondary prevention, and early rehabilitation [5, 6]. Consequently, the use of thrombolysis as reasons for non-transfer represents a potential mismatch between protocol intentions and clinical decision making in the prehospital setting.

A small number of patients were documented as being scheduled for a later appointment at the stroke unit following neurologist assessment.

Few studies have investigated the prehospital phase from the perspective of ambulance personnel and their communication with neurologists. The limited literature on prehospital neurologist consultation may partly reflect variation in EMS system organization. In many settings, suspected stroke cases are managed through prenotification of the emergency department rather than direct neurologist consultation [21], whereas the Danish prehospital system mandates neurologist involvement in destination triage.

The Stockholm Stroke Triage Study in Sweden [22] and a trial in Norway [23] have examined structured paramedic assessments combined with teleconsultation with a neurologist. Both demonstrated that direct communication improved triage accuracy and time to treatment. However, these trials tested defined triage tools or National Institutes of Health Stroke Scale (NIHSS) based assessments, unlike the current study which reflects real-world practice in a non-intervention setting. Also, a PreSS validation study in Denmark [17], showed that structured telephone conferences with a neurologists improved triage accuracy for large-vessel occlusion. In the present study, PreSS was available but not mandatory and was used inconsistently during the study period, which did not allow for meaningful analytical evaluation. Taken together, this indicates that findings from studies using structured triage interventions may not be directly transferable to routine prehospital practice, where variability in tool use and documentation is common.

Several patients (n = 97; 14%) were not documented to have been consulted with a neurologist as recommended in the prehospital stroke instructions [17]. No reason for the deviation was documented in the majority of these cases. This finding indicates either incomplete instruction adherence or insufficient documentation. A telephone consultation will always involve some degree of incomplete information, as the neurologist is not physically present with the patient. Moreover, we do not know whether the ambulance personnel consistently relay all relevant details. Neurologists base their decisions on these calls and, although they have the possibility to look up patient information, this is not always done due to time pressure. A video-assisted telephone consultation would enable the neurologist to directly observe the patient’s neurological signs and guide focused examinations, rather than relying solely on the history and assessments provided by ambulance personnel. Existing studies on video support in prehospital emergency care show inconsistent results [24, 25], and its value in stroke assessment remains uncertain. Further research is therefore needed, particularly for patients presenting with atypical or unclear symptoms.

Although documented in only one case in this study, capacity issues have been shown elsewhere to influence the assessment of admittance for thrombolysis treatment [26]. Telemedicine-based consultation could mitigate such cases by enabling real-time neurologist evaluation even when stroke unit beds are unavailable [27].

Previous studies have shown that patients presenting with few or atypical symptoms, such as confusion, dizziness, or visual disturbances, pose challenges in prehospital stroke assessment [28–30]. In the present cohort, unclear or non-specific symptoms were frequent, including sensory disturbances, gait instability, and visual complaints. These symptom presentations may complicate assessment and triage for ambulance personnel and consulting neurologists. Importantly, as the present study included only patients with dispatcher suspected stroke, it cannot address symptom presentations among patients not initially recognized as potential stroke cases at dispatch. Stroke mimics, particularly alcohol intoxication, can also complicate recognition, as overlapping symptoms like slurred speech and gait imbalance are common [31]. The high proportion of alcohol-related ambulance responses could further complicate recognition [32]. This underscores the need for targeted training in recognizing atypical or overlapping symptoms of stroke, which could be beneficial for both ambulance personnel and neurologists [30, 33].

### Strengths and limitations

This study benefits from the use of high-quality, register-based data from the Danish healthcare system. Use of the unique CPR number allowed for accurate linkage between prehospital records with the Danish Stroke Register, which has a documented completeness of 93–95% [11].

Additionally, data collection from prehospital records was standardized through a predefined instrument in REDCap, and interrater reliability was assessed and improved multiple times. The instrument achieved a Kappa score of ≥ 0.8, which indicates a strong level of reliability and reduces the risk of subjective interpretation bias when extracting data. Interpretation bias is still possible, as the two reviewers interpreting the records may have different contextual understanding or assumptions than the ambulance personnel who created the records.

The stroke unit operates separately from the emergency department, allowing for a clearer depiction of real-world patient flow. The EMS in Region Zealand follows administrative instructions, in which ambulance personnel should consult a neurologist in all suspected stroke cases before direct admission. This makes it possible to evaluate both instruction adherence and the communication chain between ambulance personnel and neurologists.

This study relied on prehospital documentation created for clinical purposes and not with the level of detail and standardization typically required for retrospective research. Frequent incomplete prehospital documentation made it difficult to determine whether missing information reflected absent findings or lack of recording. Reasons for non-admission were documented in only 34% of cases consulted with a neurologist. This likely reflects limitations of prehospital documentation in acute settings, where clinical priorities and time constraints may reduce the level of detail recorded. Consequently, absence of documentation should not be interpreted as absence of clinical reasoning.

It could also reflect insufficient emphasis on documenting decision rationale and improving structured documentation of neurologist consultation and triage decisions may enhance quality assurance and provide better insight into prehospital decision-making in suspected stroke cases.

In contrast, determining whether a neurologist had been contacted, was not influenced by missing data, as this information was more consistently registered.

An important opportunity for future work is the inclusion of neurologists’ consultation notes, which are routinely generated for all patients. Integrating these notes would make it possible to compare the neurologist’s clinical reasoning with the information transmitted by ambulance personnel and to explore how specific details influence referral decisions. Future studies should also compare symptom profiles and triage pathways for patients transported directly and those who were not suspected to have stroke by the dispatchers. These patients may differ in symptom presentation and represent an important group for future studies.

## Conclusion

While most patients were consulted with a neurologist, a notable proportion were not, despite consultation being mandatory in the prehospital protocol. Patients not consulted with a neurologist were more likely to present with vague or atypical stroke symptoms. These findings underscore the diagnostic complexities inherent in prehospital stroke assessment and point to the need for enhanced training in recognizing atypical stroke presentations, as well as a reconsideration of current triage guidelines.

## Data Availability

Due to Danish data protection laws, the use of registry-based data on Danish residents requires prior approval. Consequently, the data underlying this study cannot be shared publicly. Access may be granted to researchers upon reasonable request, and only after the relevant approvals have been obtained from the appropriate authorities.

## Abbreviations

CAD: Computer-Aided Dispatch
CPR: Civil Personal Register
EMS: Emergency Medical Services
NIHSS: National Institutes of Health Stroke Scale
PreSS: Prehospital Stroke Score
REDCap: Research Electronic Data Capture
TIA: Transient Ischemic Attack
